# The Designs and Testing of Biodegradable Energy-Absorbing Inserts for Enhanced Crashworthiness in Sports Helmets

**DOI:** 10.3390/ma17174407

**Published:** 2024-09-06

**Authors:** Paweł Kaczyński, Mateusz Skwarski, Anna Dmitruk, Piotr Makuła, Joanna Ludwiczak

**Affiliations:** 1 Department of Metal Forming Welding and Metrology, Faculty of Mechanical Engineering, Wroclaw University of Science and Technology, Wybrzeze Wyspianskiego 27, 50-370 Wroclaw, Poland; pawel.kaczynski@pwr.edu.pl (P.K.);; 2 Department of Lightweight Elements Engineering, Foundry and Automation, Faculty of Mechanical Engineering, Wroclaw University of Science and Technology, Wybrzeze Wyspianskiego 27, 50-370 Wroclaw, Poland; anna.dmitruk@pwr.edu.pl; 3 Department of Environmental Protection Engineering, Faculty of Environmental Engineering, Wroclaw University of Science and Technology, Wybrzeze Wyspianskiego 27, 50-370 Wroclaw, Poland; joanna.ludwiczak@pwr.edu.pl

**Keywords:** honeycomb, biodegradable polymers, material models, mechanical properties, FEM, dynamic test, crashworthiness

## Abstract

This article addresses manufacturing structures made via injection molding from biodegradable materials. The mentioned structures can be successfully used as energy-absorbing liners of all kinds of sports helmets, replacing the previously used expanded polystyrene. This paper is focused on injection technological tests and tensile tests (in quasi-static and dynamic conditions) of several composites based on a PLA matrix with the addition of other biodegradable softening agents, such as PBAT and TPS (the blends were prepared via melt blending using a screw extruder with mass compositions of 50:50, 30:70, and 15:85). Tensile tests showed a positive strain rate sensitivity of the mixtures and a dependence of the increase in the ratio of the dynamic to static yield stress on the increase in the share of the plastic component in the mixture. Technological tests showed that increasing the amount of the plasticizing additive by 35% (from 50% to 85%) results in a decrease in the minimal thickness of the thin-walled element that can be successfully injection molded by about 32% in the case of PLA/PBAT blends (from 0.22 mm to 0.15 mm) and by about 26% in the case of PLA/TPS blends (from 0.23 mm to 0.17 mm). Next, the thin-walled elements (dimensions of 55 × 55 × 20 mm) were manufactured and evaluated using a spring-loaded drop hammer. The 60 J impact energy was tested in accordance with the EN 1078 standard. The dynamic crushing test included checking the influence of the materials’ temperature (−20, 0, 20, and 40 °C) and the impact velocity. It was proven that the maximum deflection increases with increasing material temperature and an increase in the share of the plastic component in the mixture. The PLA15PBAT85 blend was selected as the most effective material in terms of its use as an energy-absorbing liner for sport helmets. Johnson–Cook and Cowper–Symonds material plasticizing models were constructed. Their use during dynamic FE simulation provided results that were in good agreement with those of the conducted experiment.

## 1. Introduction

Energy absorption applications (e.g., in the automotive, aerospace, architecture, sport and leisure, and biomedical sectors) utilize various kinds of materials, such as composites, hybrid materials, polymer or metallic foams, cellular structures like honeycombs, and complex hierarchical systems [1,2,3,4]. Multi-cell thin-walled systems are the ones most commonly applied [5]. Usually, the overall aim is for the material to be durable and possess a low mass simultaneously, which increases the necessity to rely on polymers or lightweight metal alloys. Moreover, such elements should be affordable regarding their production and exploitation costs. Thin-walled cellular shapes not only have low mass but also tailorable mechanical and energy absorption properties, adjustable via their variable cell size, orientation, cross-section, or wall thickness. Such materials, often applied as cores in a sandwich arrangement, exhibit various destruction mechanisms depending on their orientation toward the working load. For example, honeycombs compressed in a direction perpendicular to their walls (in-plane compression) behave quite similarly to metallic foams, as their deformation proceeds based on the local densification, up to the point where the open cells are no longer present and the structure starts to perform as a solid (homogeneous) body [6,7,8]. The main difference between these structures is the fact that the pores of foam collapse rather evenly in the whole volume, while honeycombs tend to deform gradually—e.g., in a row-by-row sequence [9]. On the other hand, if they are aligned to the main direction (out-of-plane compression) of the cells’ elongation, honeycombs act differently—their energy absorption performance is the best because of the enhanced load-bearing ability, especially with the use of thin-walled structures that enable plastic deformation via folding. Regular honeycombs can be turned into more advanced solutions, for example by combining them with other geometries in hierarchical shapes, making them multimaterial, irregular, or density-graded structures [10] (with changeable cell size or wall thickness). Among other bio-inspired spatial constructs, the following can be distinguished: nacre, conch shell, shrimp shell, horns, hooves, spiderweb, beetle wings, bones, bamboo, fish scales, pomelo, horseshoe, crocodile skin, etc. [1,11,12]. Structural foams of open or closed porosities are used as energy absorbers equally as often as honeycombs. Avalle et al. [13] tested the energy absorption characteristics during the static or dynamic deformation of three polymeric foams: expanded polypropylene (EPP), rigid polyurethane foam (PUR), and a blend of polyamide with modified polyphenylene and polystyrene (NORYL GTX^®^). PUR foams perform in a comparable manner to honeycombs regardless of the strain rate, and their deformation is permanent; therefore, they cannot withstand multiple impacts. The above-mentioned polypropylene- and polyamide-based foams exhibit similar behavior to each other—their performance is strongly dependent on the strain rate. Their energy absorption efficiency and absorber energy are higher in dynamic tests than in static ones and increase with the increase in the relative density of the sample.

Modern techniques are widely applied for the design and manufacture of complex shapes. For example, additive manufacturing offers the possibility to change the material during 3D printing, allowing it to print multimaterial honeycomb structures, such as those composed of ABS—poly(acrylonitrile butadiene styrene)—and thermoplastic PU (polyurethane), which have been described by Khatri et al. [14]. The proposed structures were easily tunable via controlling the thickness of particular layers. Kumar et al. [15] fabricated 3D-printed cellular structures from TPU, both with open and closed porosities, which were suggested to be used as energy absorbers in midsole shoes. Recently, auxetic structures have also drawn the attention of researchers [16]. Gunaydin et al. [17] tested the compressive and energy absorption behaviors of these structures in several material (nylon, nylon and carbon fiber composite, nylon and glass fiber composite) and found them to be even more effective than common hexagons. In addition to auxetic structures (otherwise called re-entrant structures), honeycombs with chiral architectures created via additive manufacturing from UV curable resin were also tested by Kumar et al. [18]. Anti-chiral and origami PLA—poly(lactic acid)—structures were analyzed by Mehrpouya et al. [19], focusing on maximizing energy absorption characteristics in sandwich materials. Ha et al. [20] studied circular hierarchical honeycombs characterized by improved relative stiffness, strength and energy absorption properties. Another new approach was reported by Wu et al. [21], who investigated hierarchical thin-walled structures based on space-filling Moore curves differing in relative density and order. Hybrid materials for energy absorption can also combine areas of metal foundry and plastics processing, as was described by Peixinho et al. [22], who reported a manufacturing route and performance analysis for aluminum spatial structures produced via investment casting joined with polymer (polypropylene PP or ABS) fillings. A similar solution was investigated by Diamantopoulou et al. [23], who utilized a polymer core and ceramics (alumina) as a lattice. Metallic tubes filled with cellular or foam-like polymer cores can also be used as energy-absorbing elements [24,25]. There have also been attempts to fill honeycomb cell interiors with various porous patterns. Ragab et al. [26] designed and manufactured PLA honeycombs with Voronoi tessellations as an infill via 3D printing; these honeycombs were characterized by superior mechanical and energy absorption properties in comparison to those of regular hexagonal structures. Their energy absorption, crash force efficiency, and specific energy absorption were in the ranges of 350 to 435 J, 1.42 to 1.65, and 1.60 to 1.82 J/g, respectivley. Other 3D-printed patterns (polyamide-12, polylactide, photocurable polymers), similar to Voronoi tesselations, that were tested in terms of energy absorption included Schwartz primitive, diamond, neovious, I-WP, and gyroid structures [27]. Gisario et al. [28] evaluated different cellular topologies for PLA custom-designed fittings for energy absorption and damping usage: lozenge, tetrachiral, anittetrachiral, rototetrachiral, hexachiral, rotochiral topologies. Octet-truss cells were also considered by Bolan et al. [29]. All of the listed examples highlight the necessity to use advanced cross-sections with a high level of complexity to ensure that the requirements for elements exposed to possible impacts during operation are met, which entails an inevitable need to use engage expensive, time-consuming manufacturing methods. In this paper, a contrasting approach is undertaken, aiming to simplify the structure and the production process, ensuring satisfactory mechanical and energy absorption performance by creating a dedicated polymer blend and utilizing the plastic folding deformation mechanism in thin-walled cellular constructs as cores in protective sports helmets. Biodegradability and replaceability were other key factors considered for this purpose.

Today, the development of biodegradable polymers has garnered significant attention as a promising solution to addressing the environmental concerns associated with conventional plastics. Biodegradable polymers, also known as biopolymers, are designed to break down naturally over time (i.e., a maximum of 6 months), reducing their impact on ecosystems and minimizing pollution [30]. Trials on the manufacturing of biodegradable polymeric foams composed of modified castor oil, styrene, and isobornyl methacrylate have been described by Dicks et al. [31]. The biodegradable polymeric structures proposed in this paper as replaceable cores for protective sports helmets have several advantages. First, users can easily replace them after a crash, restoring 100% of the helmet’s protective properties. Second, these structures can be disposed of by composting them. Third, they provide better protective properties because they utilize a previously unused mechanism of plastic folding instead of compression of polystyrene elements, in which the deformation mechanism is typical for foam-like materials, as polystyrene beads (cells) densify and collapse near the place of the applied load [32]. Plastic deformation, beneficial for the maximization of energy absorption, can be introduced by using buckling initiators [33] orby blending various materials, as is carried out in the proposed approach, to ensure the obtention of the desired mechanical properties. When using high-stiffness materials, the upper surface of the energy-absorbing element comes with so-called imperfections, i.e., cuts, for example, the purpose of which is to initiate the deformation process and reduce the force required for it (reducing the initial peak force). Such action prevents the global buckling of the element. It is crucial for the energy-absorbing materials to be simultaneously durable and ductile to some extent. In this work, where emphasis is put on designing material solutions to produce replaceable, biodegradable cores for protective sports helmets, this was a crucial goal. This research is focused on injection technological tests and tensile tests (static and dynamic conditions) on several composites: PLA with PBAT and PLA with TPS. The thin-walled elements are manufactured and evaluated using a spring-loaded drop hammer. The dynamic crushing test included checking the influence of the materials’ temperature and the impact velocity.

## 2. Materials and Methods

### 2.1. Determination of Plasticizing Curves

The materials used during the tests were based on a PLA matrix (PLI 005, NaturePlast Mondeville, France) with the addition of softening biodegradable plastics such as PBAT—poly(butylene adipate terephthalate) (Ecoflex F Blend C1200, BASF, Ludwigshafen, Germany)—and TPS (a starch-based biopolymer, MaterBi EF03A0, Novamont, Novara, Italy). The authors performed a thermal analysis of the tested materials in their previous work [34]. PLA has the highest melting temperature, approximately 170 °C, and a glass temperature equal to 61.6 °C, while other biopolymers are characterized by lower melting temperatures in the range of 115–122 °C. The tests did not reveal any decomposition that could have occurred during exposure to the temperatures during the polymer mixing process. Moreover, the PLA used for the tests is characterized by a semi-crystalline structure and is intended for injection processes, with an MFR of 20.3 g/10 min (180 °C; 2.16 kg). The mechanical properties of base materials are briefly presented in Table 1. The blends were prepared via melt blending using a twin-screw extruder (Haake PolyLab QC, Thermo Scientific, Waltham, Massachusetts, U.S.A.)—the parameters of the blend extrusion process are shown in Table 2. The following mass compositions were prepared: PLA50TPS50 (a blend consisting of 50% PLA and 50% TPS), PLA30TPS70, PLA15TPS85, PLA50PBAT50, PLA30PBAT70, and PLA15PBAT85. After the extrusion process, the materials were cooled at room temperature on a conveyor belt and then transported to the granulator, where they were cut into cylindrical granules with a height of approximately 3.5 mm and a diameter of 2.5 mm. Next, the granules were used to produce specimens for tensile testing and energy-absorbing structures (inserts) via injection molding with the use of a Demag Ergotech Compact 50–120 injection molding machine (Der Demag-Konzern, Düsseldorf, Germany).

**Figure 1:**
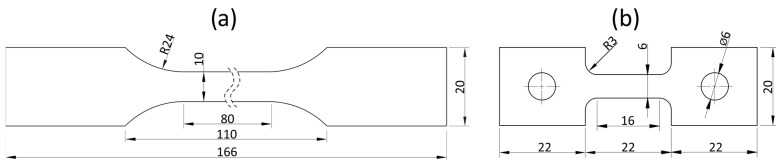
Geometry of samples used for tensile tests: (**a**) quasi-static tests; (**b**) dynamic tensile tests.

For the purpose of strength testing, 4 mm thick, flat specimens were prepared and manufactured via injection molding (parameters of the process are shown in Table 3). The typical geometry defined by the EN ISO 3167:2014 standard [35] (Figure 1a) was used for tests conducted in quasi-static conditions (strain rates of 0.01 and 0.1 s^−1^ were selected). Tensile tests were carried out on a TINIUS OLSEN H25KT testing machine (Salfords, U.K.).

**Figure 2:**
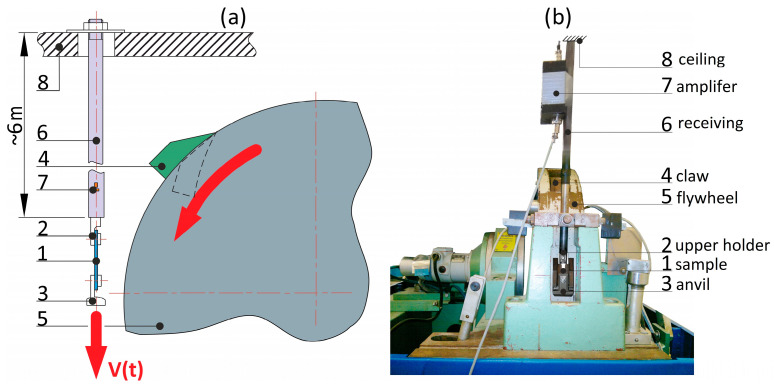
Rotary flywheel hammer: (**a**) diagram of the device; (**b**) photograph.

Samples with reduced dimensions (Figure 1b) were used for dynamic tensile tests, which allowed for high strain rates. The tests were carried out using an RSO-type rotary flywheel impact hammer manufactured by WPM Leipzig (Figure 2). The impactor’s linear velocity of 4, 7, and 14.5 m/s corresponded to the strain rate of ɛ̇ = 250, 500, and 1000 s^−1^, respectively. The force measurement methodology was based on the use of a single-rod system described by Kawata [36]. A diagram of the working part of the hammer is shown in Figure 2a. The flywheel (5) had a diameter of 0.6 m and a weight equal to 230 kg. It was precisely mounted on the shaft and accelerated by the electric motor to the desired rotational speed. Once the desired velocity was reached, the claw (4) was released by the electromagnetic lock and moved outwards by centrifugal force. The claw (4) hit the anvil (3) of the sample (1), which was mounted to the upper holder (2), connected permanently to the receiving rod (6). The rod was equipped with a dedicated measuring system (7) consisting of 1 mm long, 120 Ω foil strain gauges (4 active strain gauges glued parallel to the rod’s axis every 90° and 4 strain gauges for temperature compensation above them glued perpendicularly to rod’s axis) mounted at a distance equal to eight times the diameter of the rod from the end closer to the specimen. The selected distance guaranteed the uniform distribution of axial stresses over the entire cross-section of the rod. The other end of the rod was mounted to the ceiling (8). Data were recorded at a frequency of 1 MHz.

For each material and selected strain rate, 3–5 experiments were performed, during which force and displacement were recorded as a function of time. The obtained values were converted into engineering stress–engineering strain. The elastic part of the curves was removed, leaving only the material-plasticizing curves representing the stress to which the material had to be subjected in order to continue the deformation process for a given extent of plastic deformation.

### 2.2. Testing of Energy-Absorbing Structures

#### 2.2.1. Manufacturing

**Figure 3:**
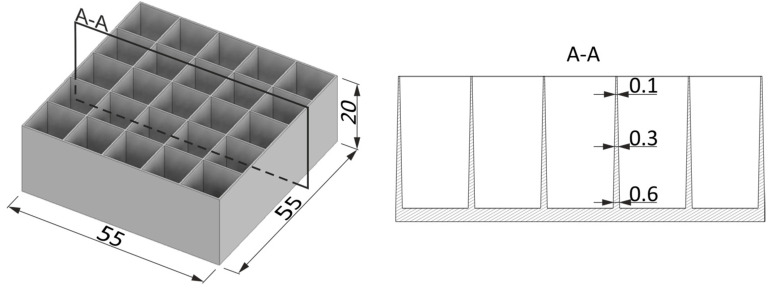
The geometry of energy-absorbing structures subjected to injection testing.

Based on the MFI (melt flow index) [34] and the plastic properties of the blends described in Section 3.1, specialized tools for evaluating the minimum gap, ensuring its complete filling during the injection process, were designed and manufactured. The tools were equipped with 25 tapered stamps and 9 inlet channels. The geometry of the empty space between stamps reflected the shape of the energy-absorbing element (Figure 3). Additionally, the slot convergence angle was set to 1.78°. The slot thickness at the narrowest point where the material was to be injected at the end was only 0.1 mm. Thin-walled, energy-absorbing honeycomb structures with a 55 × 55 mm cross-section and a height of 20 mm were manufactured. They had 11 × 11 mm pockets arranged in a 5 × 5 array. Their height was determined based on the measurement of the average height of energy-absorbing inserts made of expanded styrofoam and mounted in commercially available helmets.

**Figure 4:**
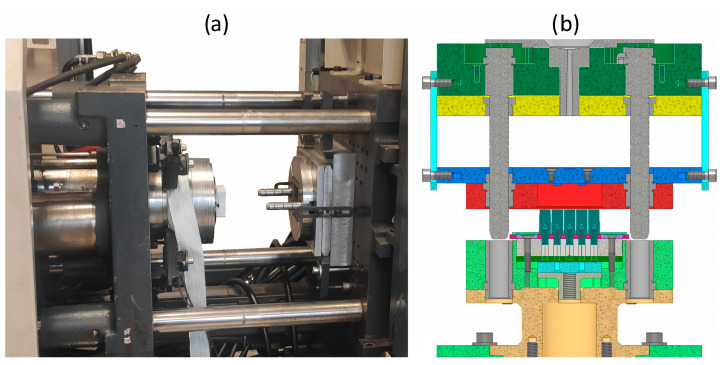
Injection mold used for the production of energy-absorbing structures: (**a**) tools mounted on the injection molding machine; (**b**) cross-section of the tools—model.

Based on manufacturing experience, it can be concluded that there is no chance of fully filling of the gaps in the described geometry. This allows for the manufacture of incompletely filled specimens. Based on sample measurements, the impact of certain manufacturing conditions (such as injection temperature, injection pressure, mold temperature) on the minimum and achievable wall thickness of the energy-absorbing insert was examined. The final design of the injection mold for producing a honeycomb structure from biodegradable plastics developed by the authors of this manuscript is under patent protection from the Patent Office of the Republic of Poland (application no. P 445650 of 21 July 2023). The tools consist of 12 movable stamps and 13 stationary stamps, as well as 4 injection points (Figure 4).

Given the very large lateral surface area of the insert, its small thickness, and the limited strength of the material, the described proprietary solution is the only way to de-mold the element without damaging it. The mold was mounted on a 50 t Demag Ergotech Compact 50–120 injection molding machine. The technological parameters of the protective insert injection process are presented in Table 4.

#### 2.2.2. Crashworthiness Testing

**Figure 5:**
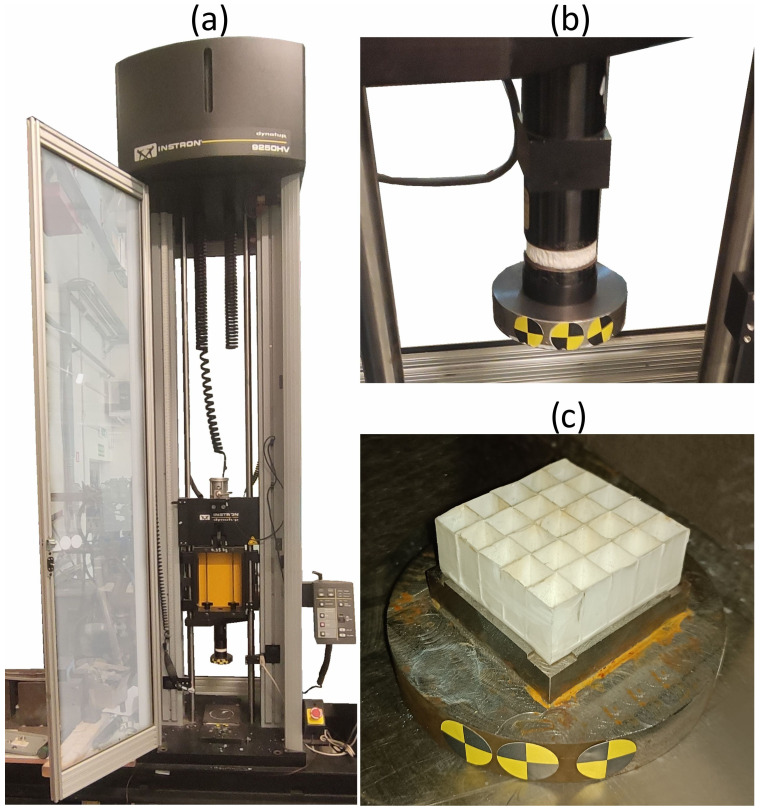
Spring-loaded dynamic crushing test stand—Instron 9250HV: (**a**) general view; (**b**) impactor’s tup; (**c**) impactor’s anvil.

Dynamic testing of the crashworthiness of the energy-absorbing structures was performed on a 9250HV Instron spring-loaded drop hammer (Massachusetts, U.S.A.), as depicted in Figure 5a. The test stand was equipped with a load cell for force signal registration (with a sampling rate of 82 kHz), while a VEO 710L Phantom high-speed camera and a dedicated image analysis software (TEMA Pro 11—Advanced Motion and Deformation Analysis Software) were used to register the high-contrast markers located before the machines’ impactor (Figure 5b) and the anvil (Figure 5c). The camera took images with an exposure of 32.5 μs plus 6.0 μs EDR at a resolution of 512 × 400 pixels (sampling rate of 31 kHz). In the next step, the sampling frequencies of both signals were unified in the FlexPro software using an advanced interpolation function, which allowed for the creation of the final force–displacement, F = f(d), graphs.

As stated in the EN-1078 standard for testing bicycle helmets, a medium-size headform (mass of 4.1 kg) manufactured according to the EN-960 standard should be used [37,38,39]. A sports helmet should be fastened on the headform and dropped freely onto the metal base from a height of 1.5 m. The kinetic energy that should be absorbed by the protective layer of the helmet should be equal to E_k_ = m·g·h = 4.1·9.80665·1.5 ≈ 60 J. During an impact, the insole located directly above the fontanel (top, central part of the skull) absorbs most of the impact energy. In order for the test conditions to be consistent with the guidelines contained in the standard, all of the manufactured inserts were subjected to 60 J impacts. An impactor weighing 8.412 kg was dropped freely onto the manufactured energy-absorbing structures from a height of 0.727 m (E_k_ = m·g·h = 8.412·9.80665·0.727 ≈ 60 J). The impact velocity was equal to V = (2·g·h)^0.5^ ≈ 3.77 m/s.

In order to test the strain rate influence, an impact velocity of 4.88 m/s (100 J) was additionally tested. To investigate the effect of the temperature on the crushing force and maximum deflection of the structure, the specimens were placed on the anvil, located in the central part of the temperature chamber (as depicted in Figure 5c). This is a very important step due to the fact that sports helmets can be used at any time of the year, so they must be resistant to low and high temperatures. The following temperatures were tested: −20, 0, 20, and 40 °C. The temperature inside the chamber was controlled with the use of an additional K-type thermocouple. The second thermocouple was attached to the specimen, thus ensuring that the measured temperature was the actual temperature of the tested material.

### 2.3. FEM Simulation

The aim of conducting this numerical simulation was to build a material plasticity model that allows for the accurate prediction of the energy-absorbing behavior of different geometries.

#### 2.3.1. Material Plasticity Model

Young’s modulus and Poison’s ratio were determined on the basis of quasi-static tensile tests, while the density was determined by measuring the dimensions and mass of the cuboid injection-molded specimens. The exact values for the selected biodegradable blend (PLA15TPS85) are as follows: Young’s modulus, 0.24 GPa; Poisson’s ratio, 0.22; density, 0.00114 g/mm^3^.

Biodegradable plastics are very complex and non-linear materials, the mechanical properties of which vary depending on a number of factors: stress level, strain rate, and temperature, among others. Therefore, in order to select a strain rate-sensitive model that best reflects the reality, the plasticization curves presented in Section 3.1 were used in order to build both Cowper–Symonds (Equation (1)) and simplified Johnson–Cook (Equation (2)) material models.

(1) $$\tilde{\sigma }=\left[1+{\left(\frac{\dot{\varepsilon }}{D}\right)}^{\frac{1}{p}}\right]$$

(2) $$\tilde{\sigma }=\left(A+B\cdot \varepsilon^{n}\right)\cdot \left[1+C\cdot ln\frac{\dot{\varepsilon }}{\dot{\varepsilon_{st}}}\right]$$

The quasi-static and dynamic tensile curves were converted into true stress–true strain curves and then transformed into plasticizing curves (as shown in Section 3.1). Next, they were subjected to statistical analysis to estimate the R^2^ (coefficients of determination). Next, both models were used in an explicit simulation to compare the crushing curves of the thin-walled structures with the data gathered during the experiment.

#### 2.3.2. Boundary Conditions

**Figure 6:**
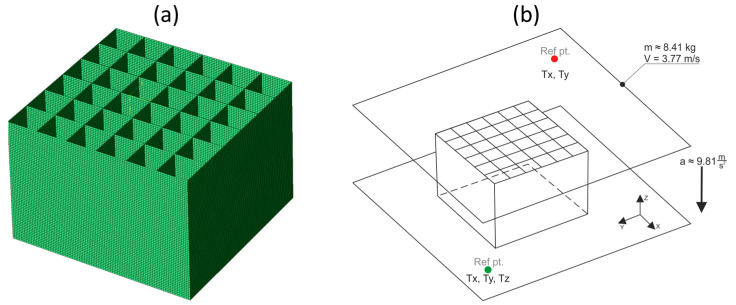
FEM model of the energy-absorbing protective insert: (**a**) mesh; (**b**) boundary conditions.

A 3D, solid geometrical model of the energy-absorbing structure was created using CATIA software. Next, a surface model was built. The middle surfaces were then exported as IGES files to FEM analysis software (ABAQUS). The imported geometry was used for mesh generation (Figure 6a). The finite model consisted of 0.5 mm 4-node, quadrilateral stress/displacement shell elements (S4R) with reduced integration and large-strain formulation.

The protective insert tested was supported at the bottom by a steel fixed plate (TX, TY, TZ) of infinite stiffness. The specimens were positioned on top of the bottom plate. A friction coefficient of 0.3 was assigned. The drop hammer’s tup that was used during the experiment was guided, only allowing movement in the vertical direction. Therefore, the tup was represented as a rigid upper plate of infinite stiffness with only one degree of freedom (TZ). The stiffness of the support and the tup was much greater than the stiffness of the plastic insert, so they can be considered perfectly rigid, without the assignation of any mechanical properties. The boundary conditions are depicted in Figure 6b.

An acceleration vector (0, 0, −9.80665) m/s^2^ was assigned to the entire finite model. A mass of 8.412 kg was assigned to the rigid point of the upper plate, which corresponded to the mass of the tup. The mass was released from a height of 0.727 m, so the initial velocity of the falling part was set to 3.77 m/s. The energy of the entire system was equal to 60 J. This energy should be absorbed (according to the EN 1078 standard concerning bicycle helmets) by the entire energy-absorbing insert of a helmet. A general contact algorithm was also applied, with the possibility of separation.

## 3. Results

### 3.1. Determination of Plasticizing Curves

**Figure 7:**
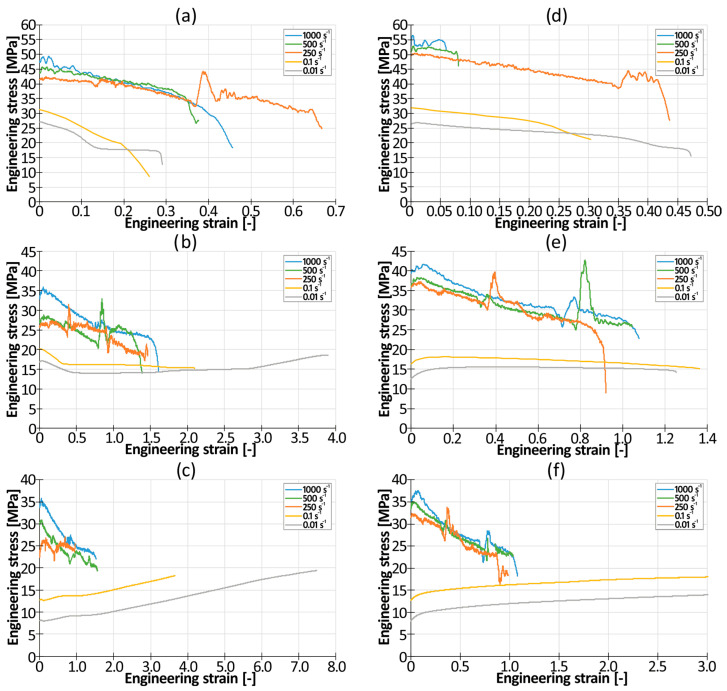
Engineering stress–engineering strain plasticizing curves of tested materials: (**a**) PLA50PBAT50; (**b**) PLA30PBAT70; (**c**) PLA15PBAT85; (**d**) PLA50TPS50; (**e**) PLA30TPS70; (**f**) PLA15TPS85.

The engineering stress–engineering strain plasticizing curves (without elastic range) obtained during the quasi-static and dynamic tensile tests are shown in Figure 7a–c (PLA/PBAT mixtures) and in Figure 7d–f (PLA/PBS mixtures). To increase the readability of the graph, only one representative curve is presented for each strain rate. Irregular oscillations of the curves recorded during dynamic testing resulted from the reflection of the elastic wave from the end of the rod that was attached to the ceiling.

Irrespective of the tested material, a significant difference between static and dynamic testing conditions could be observed. First, significant, positive strain rate sensitivity was observed. The intensity of this effect was strongly dependent on the amount of softening additive (PBAT or TPS). In the presence of 50% of this additive, the ratio of the dynamic yield strength to the quasi-static yield strength varied from 1.5 (for PLA50PBAT50) to 1.73 (for PLA50TPS50). As the amount of softening additive increased, the coefficient increased too. When the amount of softening additive reached 85%, the aforementioned coefficient was equal to 2.5 (for PLA15PBAT85) and 3.1 (for PLA15TPS85).

It could also be observed that there was a significant influence of the softening additive on the plastic properties of the blend in quasi-static conditions. An increase in the amount of the softening agent from 50% to 85% resulted in a significant increase in the elongation at break from 0.025 (for PLA50PBAT50; ɛ̇ = 0.01) and 0.35 (for PLA50TPS50; ɛ̇ = 0.01) to about 3.7 (for PLA18PBAT80; ɛ̇ = 0.01) and 3.0 (for PLA15TPS85; ɛ̇ = 0.01).

Moreover, the characteristics of the stress–strain curve were also influenced by the amount of pure PLA in the blend. For 50/50 blends, the quasi-static stress–strain curves always had a descending character. A decrease in the amount of PLA to 30% resulted in an almost constant level of tensile stress throughout the test until failure. In the case of mixtures containing only 15% of PLA, the curves always increased monotonically until failure occurred.

### 3.2. Testing of Energy-Absorbing Structures

#### 3.2.1. Manufacturing

**Figure 8:**
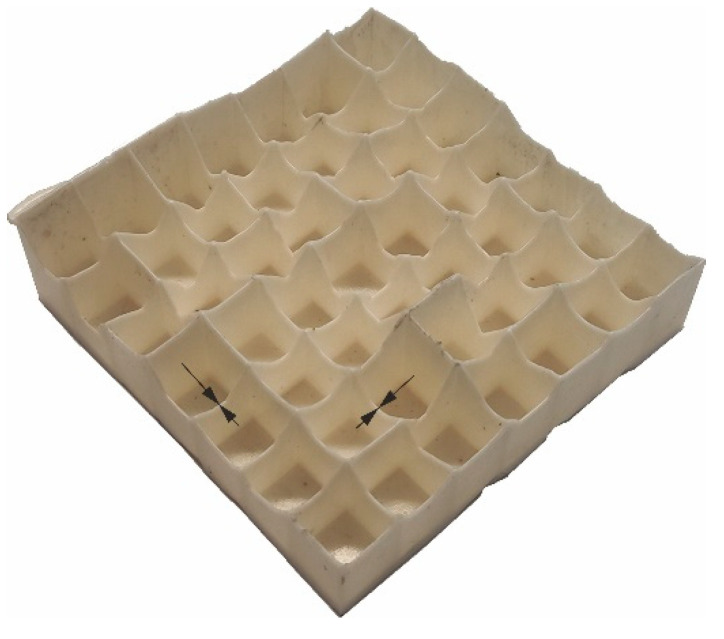
Location of measuring points for calculation of minimal thickness for successful injection molding.

The walls of the energy-absorbing structures obtained during injection tests were measured in 10 of the thinnest cross-sections located at the top that were randomly selected, as shown in Figure 8. Next, the average value was calculated. In this way, the minimum gap that could have been filled by particular blends of biodegradable plastics was determined. The measured values are presented in Table 5.

In the case of both tested combinations of materials, it can be concluded that the increase in the percentage of plasticizing additive (PBAT or TPS) in the blend resulted in a decrease in the thickness of the gap that could be successfully filled during injection molding (Table 5). Increasing the amount of plasticizing additive by 35% (from 50% to 85%) resulted in a decrease in the thickness of the gap of about 32% in the case of PLA/PBAT blends (from 0.22 mm to 0.15 mm) and of about 26% in the case of PLA/TPS blends (from 0.23 mm to 0.17 mm).

#### 3.2.2. Crashworthiness Testing

**Figure 9:**
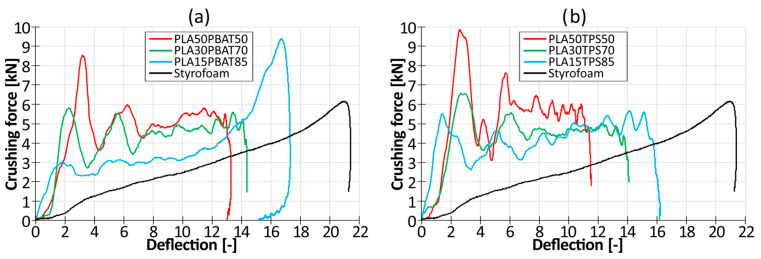
Force–deflection graphs of inserts made of blends based on (**a**) PLA and PBAT; (**b**) PLA and TPS.

Force–deflection graphs obtained during the dynamic crushing tests on the specimens ready-made at room temperature are depicted in Figure 9. A crushing curve of expanded polystyrene cut out from a commercially available bike helmet is presented as a reference.

Analyzing the force–displacement curves in Figure 9, it can be observed that during the dynamic crushing of the designed protective inserts made of bioplastics, there was a large and unfavorable force peak at the beginning of the graph, especially for materials with a higher content of PLA. This is due to the high initial resistance of the structure, which is much stiffer than an insert made of expanded polystyrene. The subsequent oscillations of the curves are related to the formation of plastic folds, which make up the most effective energy absorption mechanism. This is evidenced by the fact that the 60 J impact was absorbed by most of the tested inserts through an 11–14 mm deflection of the insert, which was over 40% smaller than that of a styrofoam insert (21 mm deflection).

The final increase in the force value on the styrofoam curve is related to the maximum and complete compression of the insert, which is associated with a high risk of complete crushing of the energy-absorbing elements. This entails increased head injuries caused by the users’ head making contact with the hard outer shell, especially for impacts of a higher energy than that defined in the standards. It can be observed that as the content of plastic PBAT or TPS increases, the curve’s oscillations are reduced and the curves begin to resemble the styrofoam crushing curve.

**Figure 10:**
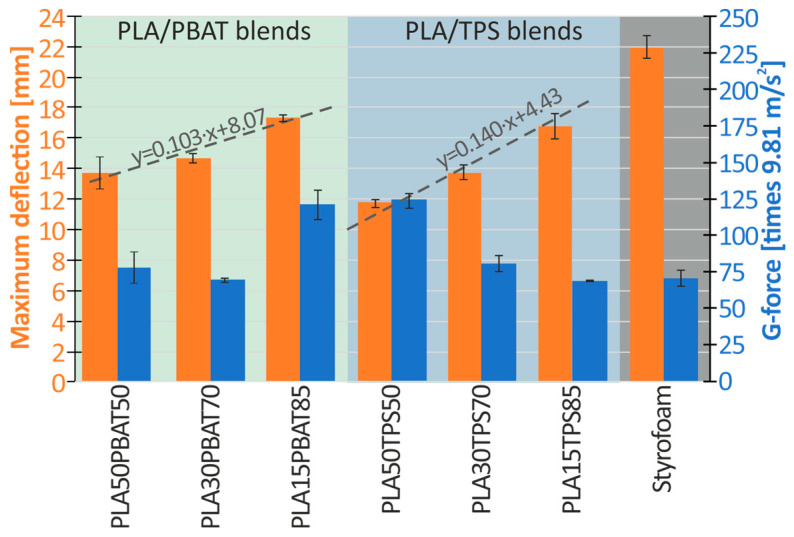
A graph of the maximum deformation of the inserts and the maximum overload occurring during crushing.

The obtained values of the maximum deflection of structures and the maximum overload (g-force) that were registered for each of the tested materials are shown in Figure 10. The trend line for individual blends is marked with a dashed line.

Based on Figure 10 (blue bars), it can be observed that none of the tested materials (biodegradable or polystyrene) exceeded the maximum permissible values defined by the EN 1078 standard (250 g, which for the impactor’s mass of 8.412 kg results in 20.6 kN).

Comparing PLA/PBAT blends with the reference styrofoam (with a deceleration of 74 g and a maximum deflection of 21.3 mm), the PLA50PBAT50 blend was characterized by a 15% higher g-force but 38% lower deflection. The PLA30PBAT70 had nearly the same g-force, but still about 33% lower deflection, while the PLA15PBAT85 blend proved to have a 54% higher acceleration level and only about 19% lower deflection. When comparing the PLA/TPS blends with the reference styrofoam, the PLA50TPS50 blend was the worst one, reaching an approximately 64% higher g-force. The most promising materials were PLA30TPS70 (35% lower deflection; 10% higher g-force) and PLA15TPS85 (24% lower deflection; 10% lower g-force). Due to the very large force peak occurring in the initial stage of crushing of the PLA50PBAT50 and PLA50TPS50 inserts, they were excluded from further research work. Such a high force peak at the beginning of an impact may cause discomfort and result in increased injuries to the user of a helmet equipped with such a protective insert.

**Figure 11:**
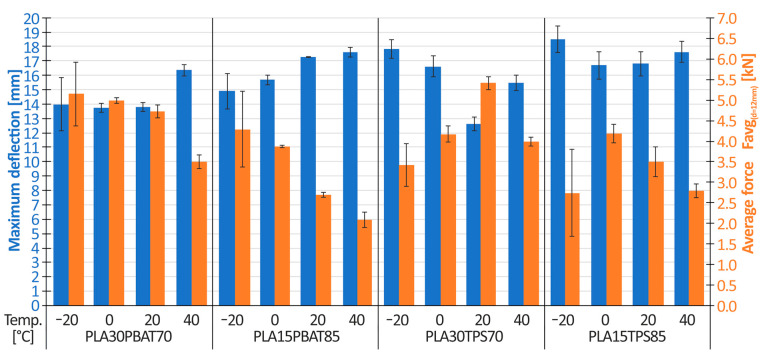
The influence of temperature on the average force (at a deflection of 12 mm) and on the maximum deflection of the energy-absorbing structures.

For the remaining materials, PLA30PBAT70, PLA15PBAT85, PLA30TPS70, and PLA15TPS85, crushing tests were performed using a temperature chamber to examine the effect of temperature on the energy absorption and maximum deflection of the structure. Results are shown in the form of a scatter chart in Figure 11. The average force for the deflection of 12 mm is marked in orange, while the maximum deflection after absorbing the entire impact energy (60 J) is marked in blue.

Analyzing Figure 11, the following conclusions were drawn:

1. There is an evident influence of the amount of plasticizer in the case of PLA/PBAT mixtures on the material performance. The PLA15PBAT85 mixture has approximately 7% (for T = −20 °C), 14% (for T = 0 °C), 25% (for T = 20 °C), and 8% (for T = 40 °C) greater deflection relative to that of the PLA30PBAT70 mixture. For both mixtures, the maximum deflection increases as the material temperature increases.
2. Samples made of the PLA30PBAT70 mixture achieved a higher average crushing force, Favg_(d=12mm)_ (average force), at a deflection of 12 mm, compared to samples made of the PLA15PBAT85 material. The differences intensified as the temperature increased. The ratios of the average force, Favg_(d=12mm)_, of the PLA30PBAT70 material to the average force, Favg_(d=12mm)_, of the PLA15PBAT85 material are 1.21 (for T = −20 °C), 1.35 (for T = 0 °C), 1.75 (for T = 20 °C) and 1.59 (for T = 40 °C).
3. Different characteristics of TPS and PBAT softening additives were noticed. In the case of temperatures ranging from −20 °C to 0 °C, comparing the same amount of TPS and PBAT additives (PLA30TPS70 vs. PLA30PBAT70 and PLA15TPS85 vs. PLA15TBAT85), materials with the addition of TPS had severalfold greater deflection and a lower average crushing force, Favg_(d=12mm)_, compared to materials based on PBAT. In the case of temperatures ranging from 20 °C to 40 °C, the opposite situation occurred: materials with the addition of TPS were characterized by lower maximum deflection and a greater crushing force.

**Figure 12:**
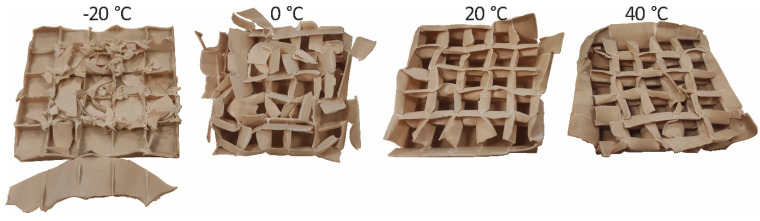
Typical crushing mode of PLA30TPST70 and PLA15TPS85.

As a result of visual inspection of the deformation mode, all of the PLA/TPS blends were rejected. Specimens made of those blends cracked brittlely regardless of the amount of TPS added, which is depicted in Figure 12.

**Figure 13:**
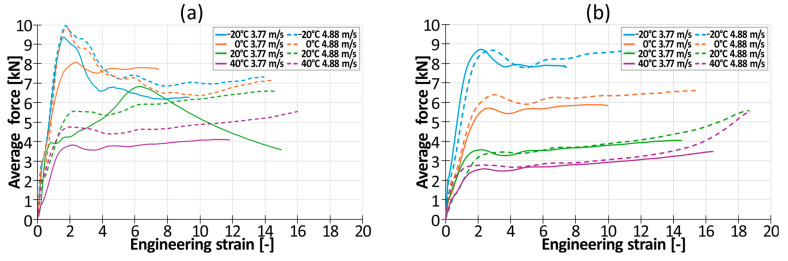
Average crushing force–deflection of selected structures: (**a**) PLA30PBAT70, (**b**) PLA15PBAT85.

**Figure 14:**
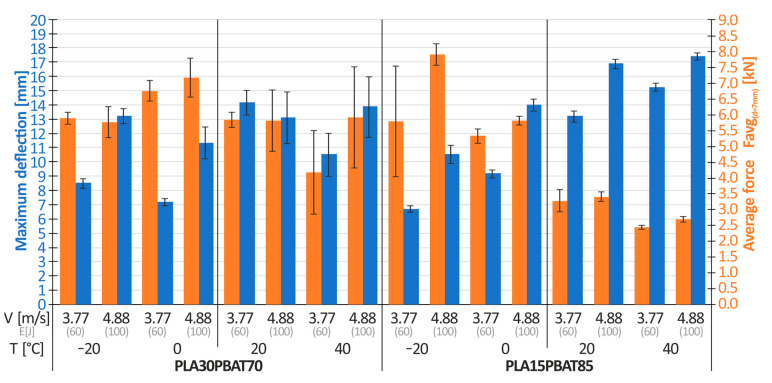
The influence of temperature on the average force (at a deflection of 7 mm) and on the maximum deflection of the energy-absorbing structures.

The PLA30PBAT70 and PLA15PBAT85 blends were selected as the most promising ones in terms of further use as energy-absorbing liners in bicycle helmets. In order to check the strain rate influence of the selected blends, two different impact velocities were applied: 3.77 m/s (60 J) and 4.88 m/s (100 J). The average crushing force–deflection curves are depicted in Figure 13, while a pivot table presenting the average crushing force at a deflection of 7 mm Favg_(d=7mm)_ and the maximum deflection is shown in Figure 14.

Based on Figure 14, it can be observed that as the impact velocity increases, the values of the average crushing force, Favg_(d=7mm)_, and the maximum deflection of the sample increase. The exception is the PLA30PBAT70 blend crushed at −20 °C and 20 °C. The average increase in the average crushing force for both materials (for all temperatures) was 13%, while the average increase in the maximum deflection was 36%. This is a very favorable phenomenon, proving that the material is not sensitive to the strain rate and, therefore, that the material maintains a similar stiffness as the impact velocity increases. The increased amount of energy is absorbed by the increased deflection of the insert. Due to this, the increased impact velocity does not cause a proportional increase in head injuries to the user of the helmet equipped with the tested inserts, and it remains at a similar level despite the increase in the impact energy from 60 J to 100 J, which is a value 66% higher than the load defined by the EN 1078 standard for testing sports helmets.

**Figure 15:**
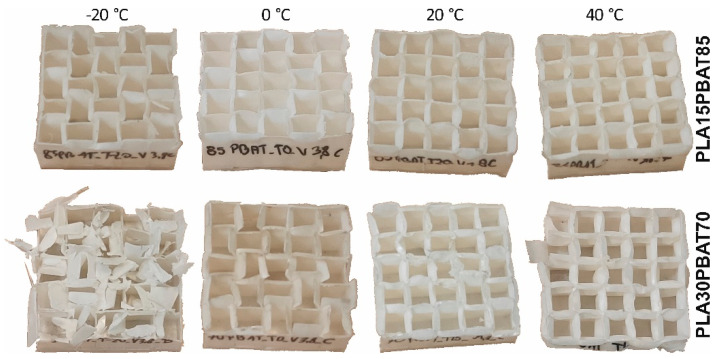
The influence of temperature on the deformation mode of specimens at an impact velocity of 3.77 m/s.

The deformation mode of the samples is depicted in Figure 15. It can be seen that the PLA30PBAT70 material at negative temperatures has a high tendency to disintegrate and is associated with defragmentation at −20 °C.

**Figure 16:**
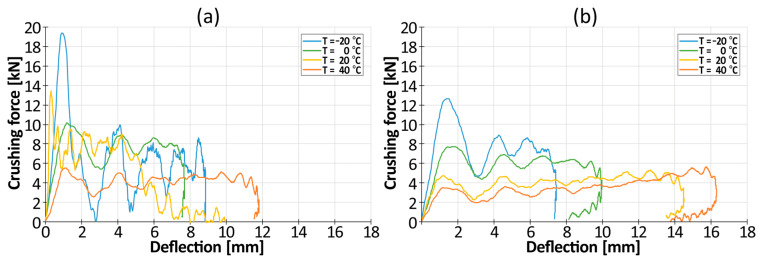
The influence of temperature on crushing force–displacement curves of (**a**) PLA30PBAT70; (**b**) PLA15PBAT85.

Moreover, at sub-zero temperatures, a large initial force peak is present, which results from the higher initial stiffness of the undeformed material. After the initiation of the folding process, the force value drops to almost 0 (Figure 16), which induces brittle cracking. This phenomenon was not observed in the case of the PLA15PBAT85 mixture. The registered values force also have about 30–50% lower oscillations. Therefore, PLA15PBAT85 is recommended for further development in sports helmets.

### 3.3. FEM Simulation

#### 3.3.1. Material Plasticity Model

**Figure 17:**
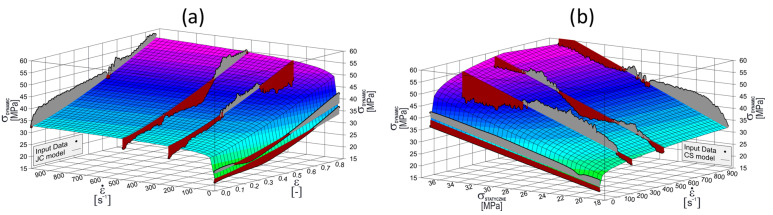
Correlation of the material plasticity models to the experimental data: (**a**) Johnson–Cook simplified model; (**b**) Cowper–Symonds model.

Based on the data recorded during the quasi-static and dynamic tensile tests on the injected dog-bone specimens, material plasticity models taking into account strain rate sensitivity (the Cowper–Symonds model and simplified Johnson–Cook model) were developed for PLA15PBAT85. The fit of the resulting Cowper–Symonds models and the simplified Johnson–Cook model to the data obtained via measurement is presented in Figure 17.

As a result of the analysis, the crucial parameters of both material models were estimated. The statistical data were also analyzed, particularly the correlation coefficient, R^2^, and confidence intervals for the significance level α = 0.05 (95% confidence level). All of the data are presented in Table 6.

The material plasticity models are characterized by a correlation coefficient, R^2^, of around 0.9, which indicates a good fit for the measurement data to the constitutive equations of the models. The simplified Johnson–Cook material model provides a much better fit than the Cowper–Symonds material model. Therefore, it is recommended to use the simplified Johnson–Cook model for the selected blend.

#### 3.3.2. Numerical Simulation of Dynamic Compression Test of Energy-Absorbing Structures

**Figure 18:**
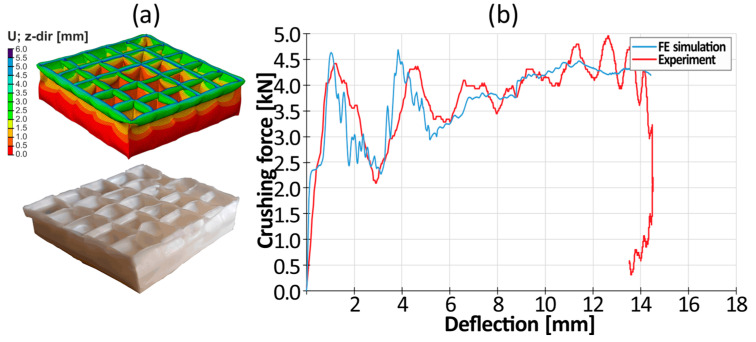
Comparison of the simulation and the crushing experiment (dynamic conditions): (**a**) deformation mode; (**b**) crushing force–deflection curve.

A comparison of the final deformation mode obtained after dynamic crushing with that in the FE simulation is depicted in Figure 18, and the force–deflection curves are presented in Figure 18b.

The simulation results turned out to be consistent with those of the experiment. The construction of the model and the numerical simulation allowed for the testing of geometric parameters such as the shape of the mesh, wall thickness, and the height of the structure. The developed numerical simulation allowed us to determine the optimal geometric parameters with high accuracy, including the appropriate ratio of the wall thickness to the height of the structure that will prevent global buckling and allow for plastic folding, which is one of the most effective energy absorption mechanisms. No significant differences were spotted between the use of the Johnson–Cook and Cowper–Symonds material models.

## 4. Conclusions

To sum up our findings, based on the results obtained from the experimental tests and numerical simulations, the following conclusions can be drawn:

1. In the case of materials’ mechanical properties, a significant, positive strain rate sensitivity was observed. The intensity of this effect was strongly dependent on the amount of softening additive (PBAT or TPS). In the case of 50% of additive, the ratio of the dynamic yield strength to quasi-static yield strength varied from 1.5 (for PLA50PBAT50) to 1.73 (for PLA50TPS50). As the amount of softening additive increased, the coefficient increased to 2.5 (for PLA15PBAT85) and 3.1 (for PLA15TPS85). An increase in the amount of the softening agent from 50% to 85% resulted in a significant increase in the elongation at break from 0.025 (for PLA50PBAT50; ɛ̇ = 0.01) and 0.35 (for PLA50TPS50; ɛ̇ = 0.01) to about 3.7 (for PLA18PBAT80; ɛ̇ = 0.01) and 3.0 (for PLA15TPS85; ɛ̇ = 0.01).
2. There is a clear influence of the amount of plasticizer in the case of PLA/PBAT energy-absorbing structures. The PLA15PBAT85 mixture had an approximately 7% (for T = −20 °C), 14% (for T = 0 °C), 25% (for T = 20 °C), and 8% (for T = 40 °C) deflection relative to that of the PLA30PBAT70 mixture. For both mixtures, the maximum deflection increased as the material temperature increases.
3. The results of both the Johnson–Cook and Cowper–Symonds material models are in good agreement with those of the experiment. This allows for the further prediction of optimal geometric parameters of energy-absorbing structures on the basis of FE simulations.
4. In the case of both tested combinations of materials, it can be concluded that the increase in the percentage of plasticizing additive (PBAT or TPS) in the blend resulted in a decrease in the thickness of the gap that could be successfully filled during injection molding (Table 5). Increasing the amount of the plasticizing additive by 35% (from 50% to 85%) resulted in a decrease in the thickness of the gap by about 32% in the case of the PLA/PBAT blends (from 0.22 mm to 0.15 mm) and by about 26% in the case of the PLA/TPS blends (from 0.23 mm to 0.17 mm).
5. As the content of plastic PBAT or TPS increases, the curve becomes flatter, i.e., the amplitude representing the formation of the plastic folds becomes smaller, and the curves begin to resemble a styrofoam curve.

## Figures and Tables

**Table 1:** Material properties of PLA, PBAT, and TPS.

Material	Ultimate Tensile Strength [MPa]	Maximum Elongation [%]
PLA	60–70	7–8
PBAT	14–20	570–740
TPS	12–15	560–580

**Table 2:** Parameters of the blend extrusion process.

Material	Temperature in Subsequent Zones [°C]	Screw Speed [rad/s]
PLA50PBAT50	155-160-180-170	5.24
PLA30PBAT70	150-155-165-150	5.24
PLA15PBAT85	155-155-160-150	4.19
PLA50TPS50	150-160-165-155	5.24
PLA30TPS70	150-155-165-155	3.14
PLA15TPS85	150-155-160-150	2.09

**Table 3:** Parameters of the sample injection molding process.

Material	Temp. in Subsequent Zones [°C]	Max. Injection Pressure [MPa]	Clamping Pressure [MPa]
PLA50PBAT50	170–190	200	70
PLA30PBAT70	160–180	170	70
PLA15PBAT85	160–180	150	70
PLA50TPS50	170–190	200	70
PLA30TPS70	160–180	170	70
PLA15TPS85	160–180	150	70

**Table 4:** Technological parameters of the injection molding process.

Blend	PLA/PBAT Blend	PLA/TPS Blend
Mold temperature [°C]	50	50
Injection molding screw temperature [°C]	205-200-190-180	210-205-195-185
Injection pressure [bar]	900	950
Injection velocity [m/s]	120	120
Injection time [s]	8	8
Clamping pressure [bar]	130	130
Cooling time before opening the mold [s]	40	40
Injected volume [cm^3^]	20	20

**Table 5:** The sizes of the minimum gaps that can be filled by the tested materials.

Material (PBAT Blends)	Minimal Gap [mm]	Material (TPS Blends)	Minimal Gap [mm]
PLA50PBAT50	0.22 ± 0.01	PLA50TPS50	0.23 ± 0.01
PLA30PBAT70	0.18 ± 0.02	PLA30TPS70	0.20 ± 0.02
PLA15PBAT85	0.15 ± 0.01	PLA15TPS85	0.17 ± 0.02

**Table 6:** Crucial coefficients of material plasticity models.

PLA15PBAT85 Material Plasticity Model	Param.	Value [-]	Σ [-]	Value [-]	R^2^ [-]
Cowper–Symonds $\tilde{\sigma }=\left[1+{\left(\frac{\dot{\varepsilon }}{D}\right)}^{\frac{1}{p}}\right]$	D p	5647 4.85	D_0.95_ p_0.95_	±432 ±0.14	0.88
Johnson–Cook simplified. $\tilde{\sigma }=\left(A+B\cdot \varepsilon^{n}\right)\cdot \left[1+C\cdot ln\frac{\dot{\varepsilon }}{\dot{\varepsilon_{st}}}\right]$	A B C n	19.90 17.27 0.0565 1.2601	A_0.95_ B_0.95_ C_0.95_ n_0.95_	±0.21 ±0.23 ±0.0008 ±0.0451	0.92

## Data Availability

The data presented in this study are available on request from the corresponding author due to patent protection and commercial nature of the results.
